# Neuroblastoma Masquerading as Constipation: Reducing Diagnostic Error With the Pediatric Abdominal Point-of-Care Ultrasound Survey

**DOI:** 10.7759/cureus.74427

**Published:** 2024-11-25

**Authors:** Jonathan Schonert, Nick Denne, Joe Minardi

**Affiliations:** 1 Department of Emergency Medicine, St. Luke's Hospital, Chesterfield, USA; 2 Department of Emergency Medicine, West Virginia University School of Medicine, Morgantown, USA

**Keywords:** abdominal neuroblastoma, bedside ultrasound, emergency medicine ultrasound, neuroblastoma, paps, pediatric ultrasound, pocus, pocus (point of care ultrasound), ultrasound

## Abstract

Abdominal pain is a common pediatric complaint in the emergency department and other clinical settings. While most causes are benign, dangerous and time-sensitive conditions may be present. Point-of-care ultrasound (POCUS) is a diagnostic modality that can help more thoroughly explore the differential diagnosis at a relatively low cost, without exposure to ionizing radiation, and in a timely manner.

This report presents the case of a three-year-old male with three weeks of abdominal pain, constipation, poor appetite, and weight loss that were evaluated multiple times for presumed constipation. POCUS at a rural critical access emergency department revealed an echogenic mass encasing the aorta and causing hydronephrosis, later confirmed as neuroblastoma on CT and biopsy. Comprehensive oncologic care was initiated at a tertiary care center.

Neuroblastoma, one of the more common forms of childhood cancer, particularly in the first year of life, often presents with non-specific symptoms that can delay diagnosis. This case report highlights the role of POCUS, and the authors introduce the incorporation of the PAPS exam (pediatric abdominal POCUS survey) in the evaluation of vague pediatric abdominal pain to improve diagnostic accuracy and reduce errors.

## Introduction

Abdominal pain and constipation are frequent chief complaints in pediatric populations in the emergency department and other clinical settings. While acute presentations often raise concern for more serious conditions such as appendicitis, intussusception, or ovarian torsion, subacute or chronic symptoms are more commonly associated with benign or self-limiting causes. However, some serious conditions can present with subtle or vague symptoms where earlier diagnosis can help improve prognosis. Neuroblastoma, which accounts for 7% of all childhood cancers, is one such condition and is the most common neoplasm in the first year of life [1].

Point-of-care ultrasound (POCUS) has become more and more prevalent in the emergency department [2] and has shown promise in expediting care for certain pathologies [3]. Particularly in the pediatric population or in resource-limited settings, POCUS can help more thoroughly explore the differential or help direct care in undifferentiated abdominal pain when very often blood work or X-rays have limited roles. This case report highlights the impact of POCUS on an accurate diagnosis in a pediatric patient who had repeatedly been diagnosed and treated for constipation but was ultimately diagnosed with neuroblastoma. We introduce the PAPS exam (pediatric abdominal POCUS survey) as a rapid bedside evaluation in vague pediatric abdominal pain to improve accuracy and reduce diagnostic errors.

## Case presentation

A three-year-old male presented to a rural critical access emergency department with at least three weeks of abdominal pain, constipation, poor appetite, and weight loss. He had been evaluated multiple times at various other facilities and diagnosed with constipation treated with laxatives.

**Figure 1:**
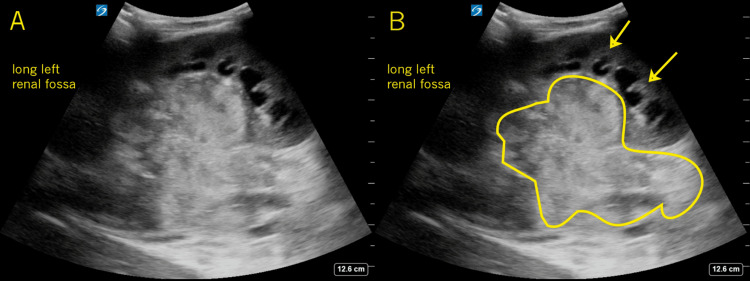
Long Left Renal Fossa In this longitudinal image from the left renal fossa, the enlarged kidney is seen (frame B arrows) with some dilation of the renal pyramids. Additionally, a large echogenic mass involving the entire renal pelvis is seen (frame B outlined). These findings should be recognized as abnormal to anyone with a basic understanding of normal sonographic anatomy.

**Figure 2:**
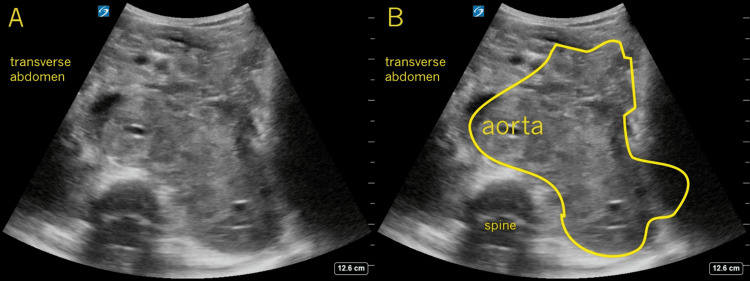
Transverse Mid-abdomen In this transverse image taken from the mid-abdomen, landmarks such as the transverse spine (frame B labeled) and the aorta (frame B labeled) can help orient the operator. A large, slightly irregular, mostly echogenic mass is identified that displaces and distorts the normal abdominal structures. Again, an operator with a basic understanding of normal sonographic anatomy should recognize the abnormal appearance of these findings.

On examination, his vital signs were normal, and he appeared pale and mildly unwell, but nontoxic and in no acute distress. He resisted abdominal examination but did not appear to have focal tenderness or peritoneal signs. The differential on history would be broad including, but not limited to, functional constipation, gastroesophageal reflux disease, gastroenteritis, diabetes, neuroblastoma, lymphoma, urinary tract infection, and appendicitis. POCUS was performed on initial evaluation, which revealed an echogenic mass arising from the left renal pelvis causing mild hydronephrosis (Figure 1 and Video 1). The mass extended into the mid-abdomen and, on subsequent views, appeared to be encasing the aorta (Figure 2 and Video 1). Emergency department laboratory findings revealed normal urine, chemistries, and mild anemia with a hemoglobin value of 8.9 g/dL.

**Video 1:** Neuroblastoma Case In this video, the images of this case presentation are presented in video form with relevant highlights.

**Figure 3:**
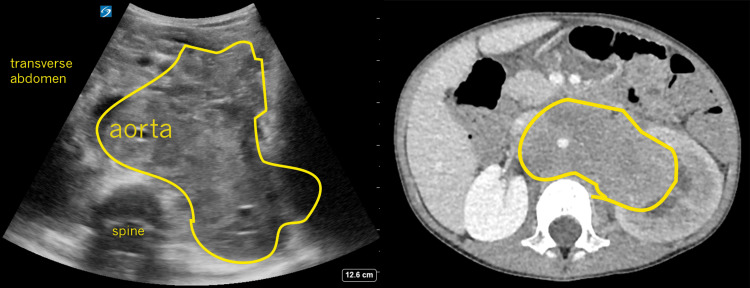
Transverse Abdomen Side-by-Side Views of Bedside Ultrasound and CT Abdomen/Pelvis Bedside ultrasound image (left) of the aorta encased in the mass arising likely from the right side which can be seen better on the CT scan (right) with the aorta lumen enhanced by contrast.

**Figure 4:**
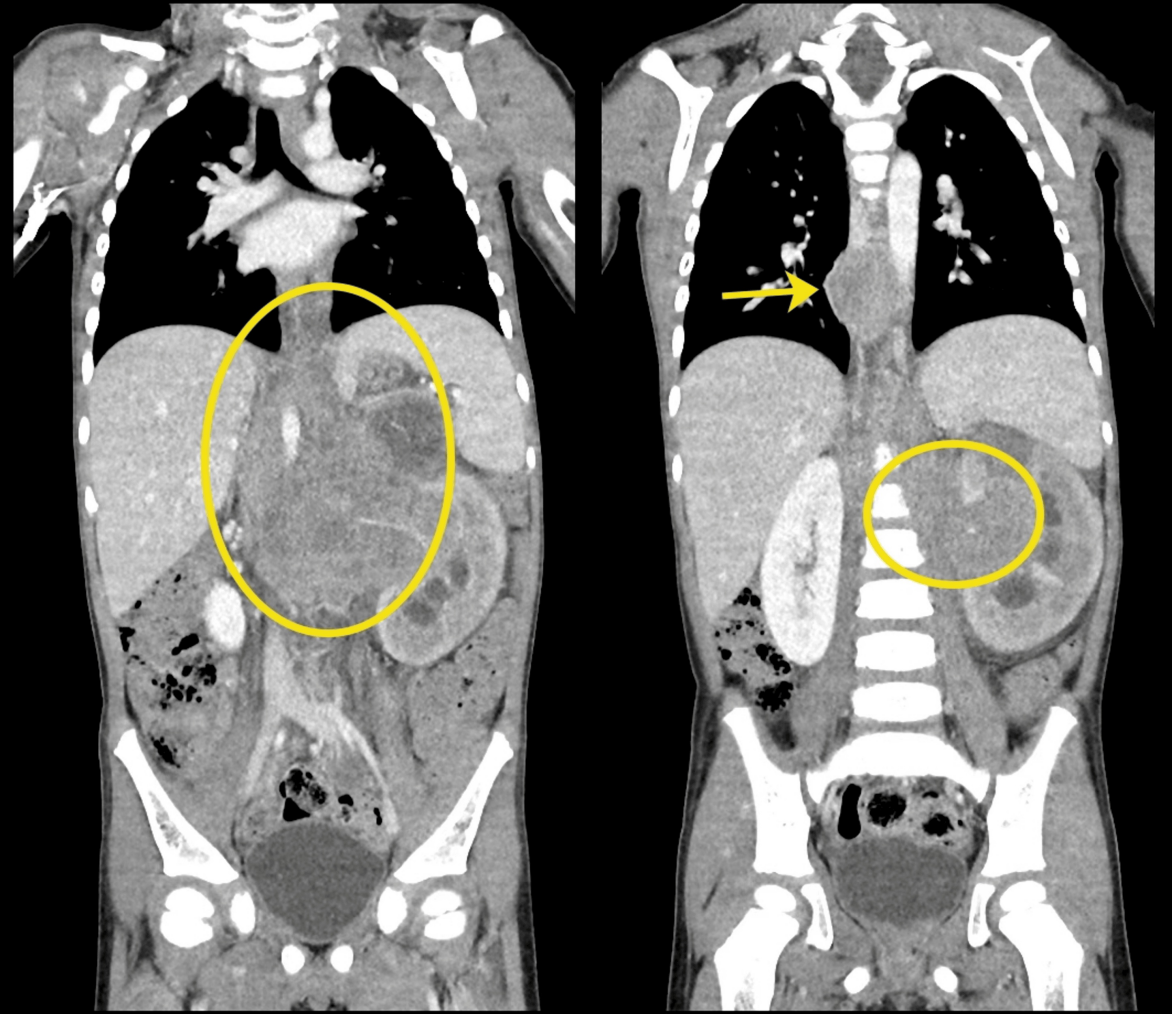
Coronal Views of CT Chest/Abdomen/Pelvis The irregular mass is seen in these coronal CT views (circled). The thoracic mediastinal lymphadenopathy is also visualized (arrow).

CT scan of the chest, abdomen, and pelvis with IV contrast was performed revealing bulky retroperitoneal lymphadenopathy displacing the aorta and the kidney with left hydronephrosis (Figures 3, 4). There was also retrocrural adenopathy in the posterior mediastinum. The patient was transferred to a tertiary care hospital with pediatric oncology capabilities. After undergoing a biopsy, he was ultimately diagnosed with a poorly differentiated metastatic neuroblastoma. Comprehensive oncologic care was initiated.

## Discussion

Neuroblastoma is a malignancy arising from primitive ganglion cells from the sympathetic nervous system. The most common primary site is the adrenal gland though they can also start anywhere along the sympathetic nervous system including other abdominal sites, the thorax and cervical regions, and pelvic ganglia [4]. Metastases are very common and present in adjacent lymph nodes, liver, bone, and skin. Since 2/3 arise from the abdomen, painless abdominal masses are a common presenting symptom or finding [5]. The expanding mass can lead to obstructive symptoms including compression of the bowel, bladder, and venous or lymphatic structures. Like other cancers, weight loss and fevers are a common sign [6]. Metastatic spread can cause bone pain/back pain, spinal cord compression, periorbital ecchymoses and Horner syndrome (orbital metastasis), and hypertension.

Neuroblastomas also do not present with any characteristic findings on routine laboratory investigations [6]. Very specific urine tests can be helpful in the complete workup for neuroblastoma due to the breakdown of catecholamines (90% of patients will have positive urine homovanillic acid and vanillylmandelic acid [7], though these aren’t readily accessible tests and have shown no benefit as a screening tool [8].

In this case, our POCUS exam noted that the left kidney appeared grossly abnormal with mild hydronephrosis and a centrally located echogenic mass that extended into the mid-abdomen. Neuroblastomas often appear as solid, heterogeneous masses on ultrasound, and can be distinguished from other abdominal tumors, such as a Wilms’ tumor, by their tendency to compress the kidney rather than originate from it. Additionally, neuroblastomas often cross the midline and can invade the surrounding vascular structures, as seen in this case where the mass encased the aorta [6].

Early identification of a neuroblastoma is critical as the prognosis depends on several factors including the age of the patient, the stage of the disease, and the tumor’s biological characteristics which would be found on subsequent biopsies. Treatment typically involves a combination of chemotherapy, surgery, and radiation therapy, with overall survival rates varying based on risk stratification [9].

The use of POCUS in this case was instrumental in identifying the presence of an abnormal mass and expedited further diagnostic imaging and treatment. Research continues to show that emergency physicians can be as accurate as ultrasonographers in critical pediatric diagnoses such as appendicitis [10], intussusception [11], and testicular torsion [12]. Applying a more liberal and broader approach to abdominal pain rather than focusing on a specific diagnosis can help prevent misdiagnosis [13] and even diagnose less common etiologies [14,15].

When performing POCUS for pediatric abdominal pain, the authors endorse a syndromic approach that considers the differential diagnosis rather than an approach based on a single organ or organ system. For instance, in patients with right-sided abdominal pain, appendicitis may be high on the differential diagnosis, but evaluation of the right kidney for hydronephrosis, and even the gallbladder can be rapidly achieved and may significantly refine and shorten the differential considerations.

**Figure 5:**
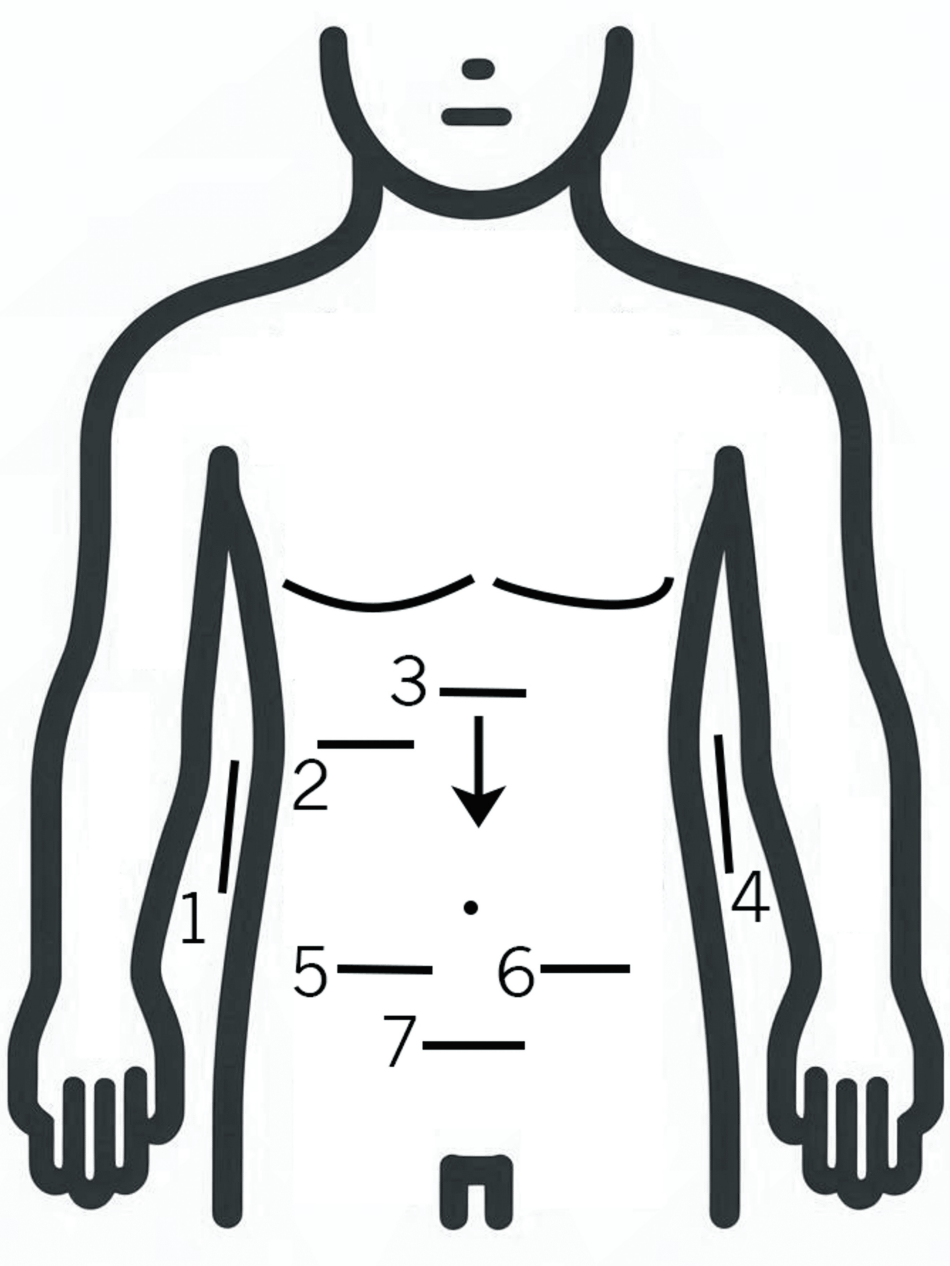
Pediatric Abdominal Survey With Point-of-Care Ultrasound Diagram 1. Longitudinal orientation in the mid-axillary line near the level of the xiphoid process and perform a broad sweep of the structures in the right upper quadrant. Coronal views of the diaphragm, liver, right kidney, and often the gallbladder can be visualized. Peritoneal fluid in the subdiaphragmatic space, Morrison’s pouch, or paracolic gutter can be seen. Additionally, any gross intestinal abnormalities in the right paracolic gutter may be visualized. 2. Transverse view in the right upper quadrant near the mid-clavicular line with wide sweeps to further evaluate the liver, its vasculature, the gallbladder, and any obvious intestinal abnormalities should be performed. Emphasis on binary recognition of grossly normal versus abnormal anatomy. 3. Transverse view of the epigastrium should be obtained, identifying the spine, aorta, and inferior vena cava as landmarks. Take note of gross pancreatic or intestinal abnormalities. 4. Longitudinal sweep of the left upper quadrant in the posterior axillary line at the level of the xiphoid, evaluating the diaphragm, spleen, left kidney, and left paracolic gutter. Gross abnormalities of the solid organs, as well as the stomach and other visible intestinal structures, may be visualized. 5. Transverse views of the right lower quadrant, identifying the iliac fossa, iliopsoas muscles, and iliac vessels as landmarks, again take note of gross abnormalities of visible intestinal structures, any masses, fluid collections, or inflammatory changes. 6. Transverse views of the left lower quadrant, identifying the iliac fossa, iliopsoas muscles, and iliac vessels as landmarks, again take note of gross abnormalities of visible intestinal structures, any masses, fluid collections, or inflammatory changes. 7. Transverse sweep of the suprapubic area, identifying the bladder as a landmark, gross abnormalities of the female pelvis should be identified in females, as well as fluid collections, masses, or visible intestinal abnormalities.

In patients with more vague or less focused abdominal pain, the authors incorporate what we refer to as the PAPS exam (as illustrated in Figure 5) to rapidly evaluate the visible solid organs (kidneys, liver, spleen, pancreas, and female pelvis where applicable), fluid-filled structures (gallbladder, urinary bladder, aorta, and inferior vena cava), and finally, the paracolic gutters and lower quadrants for fluid collections, inflammatory findings, or obvious dilated or thickened bowel loops. Our approach includes lateral longitudinal renal views, a view of the right upper quadrant, the epigastrium following the length of the abdominal aorta and inferior vena cava, a view of each lower quadrant, and finally, the pelvis. While such an approach may appear laborious and time-consuming, the necessary views can be obtained in a relatively efficient manner that is very analogous to a FAST (Focused Assessment With Sonography in Trauma) exam.

## Conclusions

Neuroblastomas, though rare, should be considered in the differential diagnosis of pediatric patients presenting with persistent abdominal pain with constitutional symptoms. Typical presentations include abdominal pain and symptoms related to mass effects from the cancer with potential metastasis.

Early and liberal integration of POCUS in the assessment of pediatric abdominal pain can help aid prompt identification of more serious conditions, reducing diagnostic error, expediting further workup, and potentially improving outcomes. Emergency physicians and others caring for children should continue to integrate POCUS and protocols like the PAPS exam into their practice and gain competence in the normal and grossly abnormal appearance of anatomical structures.
